# Cognitive dysfunction in patients with Systemic Lupus Erythematosus

**DOI:** 10.12669/pjms.331.11947

**Published:** 2017

**Authors:** Bilal Azeem Butt, Sumaira Farman, Saira Elaine Anwer Khan, Muhammad Ahmed Saeed, Nighat Mir Ahmad

**Affiliations:** 1 Bilal Azeem Butt, FCPS (Medicine). Division of Rheumatology, Fatima Memorial Hospital, Lahore, Pakistan; 2 Sumaira Farman, FRCP, FACP, FACR, SCE Rheumatology. Division of Rheumatology, Fatima Memorial Hospital, Lahore, Pakistan; 3 Saira Elaine Anwer Khan, MRCP, SCE Rheumatology. Division of Rheumatology, Fatima Memorial Hospital, Lahore, Pakistan; 4 Muhammad Ahmed Saeed, FCPS (Rheumatology) FACR, FCPS (Medicine). Division of Rheumatology, Fatima Memorial Hospital, Lahore, Pakistan; 5 Nighat Mir Ahmad, FACP, FACR, DABR, DABIM. Division of Rheumatology, Fatima Memorial Hospital, Lahore, Pakistan

**Keywords:** Systemic Lupus International Collaborating Clinics (SLICC), Systemic Lupus Erythematosus (SLE), Cognitive dysfunction, Montréal Cognitive Assessment (MoCA)

## Abstract

**Objective:**

To determine the frequency of cognitive dysfunction in patients with Systemic Lupus Erythematosus in a Pakistani population, presenting at a tertiary care Rheumatology setting.

**Methods:**

This cross-sectional study was conducted at the Division of Rheumatology, Fatima Memorial Hospital, Lahore, from March to June 2016. A total of 43 consecutive patients, who fulfilled the 2012 SLICC (Systemic Lupus International Collaborating Clinics) classification criteria for Systemic Lupus Erythematosus (SLE), were enrolled. Cognitive function was assessed using Montréal Cognitive Assessment (MoCA) questionnaire. Demographic data and disease dynamics were collected in a proforma. Cognitive dysfunction was defined as score < 26/30, adjusted for duration of formal education. SPSS version 16.0 for windows was used to analyse data and to calculate frequency of cognitive dysfunction.

**Results:**

Out of 43 enrolled patients, 95.3% were females and 4.7% were males, with mean age of 28.72 ± 9.25 years and mean formal education duration of 10.98 ± 3.29 years. The mean disease duration was 24.21 ± 30.46 months. Anti-nuclear antibodies (ANA) were present in all patients and anti-ds DNA in 93% patients. Cognitive dysfunction according to MoCA score was found in 65.1% (n=28) patients. For patients with disease duration more than two years, cognitive dysfunction was found in 60% patients [p>0.05] and for duration of formal education less than 12 years in 74.1% patients [p>0.05].

**Conclusion:**

In this study, two third of SLE patients had Cognitive dysfunction. Hence, there is an increasing need to recognise and initiate early therapy for this overlooked aspect of SLE with an aim to achieve better quality of life.

## INTRODUCTION

Systemic lupus erythematosus (SLE) is a diverse, chronic autoimmune disease presenting with manifestations encompassing almost any organ of the body.^1^ Neuropsychiatric syndromes in SLE encompass a wide spectrum, ranging from stroke, seizure and psychotic episodes to mood changes, headache and cognitive problems.^2^ American College of Rheumatology (ACR), defined 19 neuropsychiatric syndromes of neuropsychiatric lupus and divided them into 12 central nervous system disorders and 7 peripheral nervous system disorders.^3^ The ACR’s revised nomenclature emphasised on cognitive dysfunction as being a major neuropsychiatric syndrome and defined it as ‘‘significant deficit in any or all of the following cognitive functions: complex attention, executive skills (e.g., organizing, sequencing, planning), memory (e.g., recall, learning), visual-spatial processing, language (e.g., verbal fluency) and psychomotor speed’’.^3^

Several studies have postulated the mechanisms responsible for cognitive dysfunction in SLE, identifying a possible role of autoantibody activity and cerebral ischemia.^4^ Additional factors have also been suggested including: health characteristics (disease duration, disease activity and medication use), immune activity (pro-inflammatory cytokines) and behavioural factors.^5^ The role of a subset of anti-DNA antibodies has been suggested, that cross reacts with specific sequences present on ligand binding domain of NR2 receptors, more specifically NR2a and NR2b.^6^ These NR2 receptors are a subtype of N-methyl-D-aspartate (NMDA) receptors, to which glutamate binds and causes excitatory effect.^7^ These receptors have been found in high concentration in the hippocampus, which affects learning and memory.^7^ Other antibodies with pathogenic relevance to cognitive dysfunction include anti-cardiolipin antibodies (ACL), anti-neuronal antibodies, anti-endothelial-cell antibodies (AECAs) and anti- Nedd5 C-ter antibodies.^8^-^10^ Patients with systemic lupus erythematosus (SLE) who have neuropsychiatric involvement were found to have significantly higher levels of matrix metalloproteinase-9 (MMP-9) in their serum and in cerebrospinal fluid (CSF), as compared to those without neuropsychiatric syndromes.^11^,^12^

Several confounding factors have been evaluated in studies with the following conclusions; neither depression nor anxiety is the primary cause of cognitive dysfunction in SLE, also there is no relationship between cognitive decline and the use of corticosteroids and neither disease activity nor duration of SLE has any association with cognition.^13^ Cognitive dysfunction is prevalent in SLE, ranging from 12% to as high as 87% and even 95%.^3^,^8^,^13^

Despite the increased awareness among rheumatologists, the etiology, course and treatment of SLE-associated cognitive dysfunction remains elusive.^14^ Thus there is an increasing need to conduct more research on this topic to emphasise the importance of early recognition and initiation of treatment of cognitive dysfunction with an aim to improve quality of life of these SLE patients. To the best of our knowledge no local study has specifically addressed cognitive dysfunction in Pakistani patients with lupus. The aim of this study was to ascertain the frequency of cognitive dysfunction in patients with systemic lupus erythematosus in a Pakistani population.

## METHODS

This cross-sectional, observational study was conducted in Division of Rheumatology, Fatima Memorial Hospital, Lahore, from March to June 2016, after approval from Institutional Review Board (IRB), Fatima Memorial Hospital, Lahore.

A total of 43 patients were selected from both outpatient and inpatient departments after sample size calculation (95% Confidence level, 10% margin of error and taking frequency of cognitive dysfunction in Systemic Lupus Erythematosus of 87%).^3^,^8^,^13^ All these patients fulfilled the 2012 SLICC (Systemic Lupus International Collaborating Clinics) classification criteria for Systemic Lupus Erythematosus and excluded those patients who were unable to read or write and those who had uraemia, sepsis or uncontrolled thyroid disease, as these affect cognition. Written informed consent was taken from each patient for participation in study and confidentiality was maintained.

The cognitive function of these patients was assessed by using the Urdu version of Montréal Cognitive Assessment (MoCA) questionnaire (Appendix I). Prior permission was taken for its use. All patients who had score < 26/30, adjusted for education (1 score added if total years of formal education was 12 years or less), were considered as having cognitive dysfunction. Demographic variables like age, sex and years of education and disease characteristics like disease duration in months, and presence of anti-nuclear antibodies (ANA) and anti ds DNA antibodies were also noted. All this information was collected in a specially designed proforma.

**APPENDIX-I:**
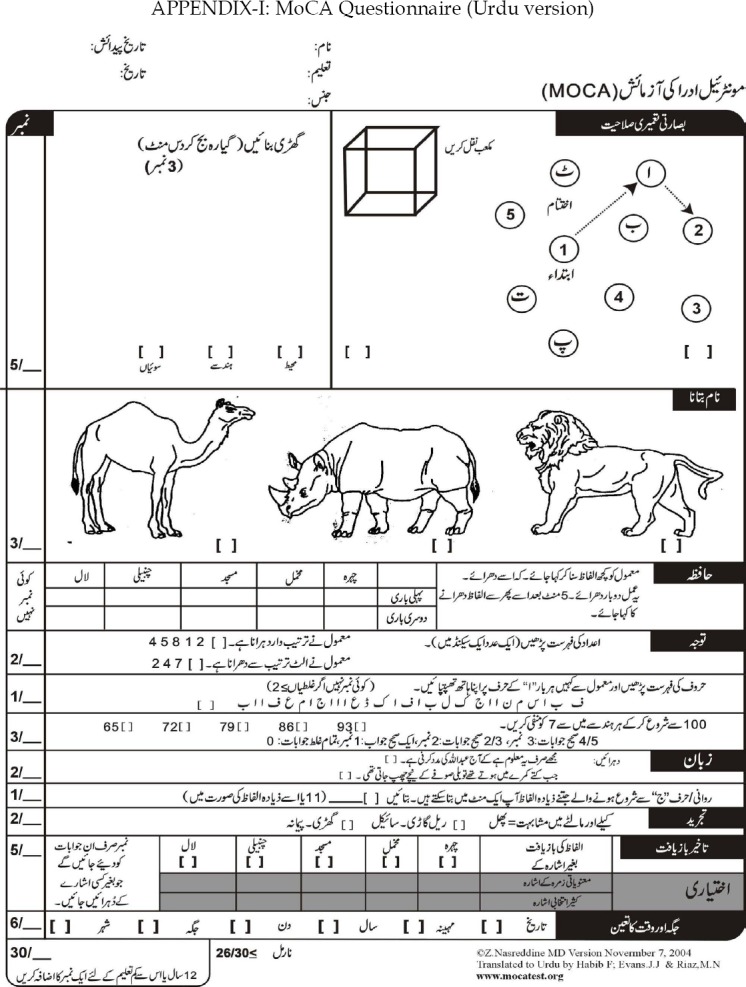
MoCA Questionnaire (Urdu version)

Data was entered in and analysed by SPSS version 16 for windows. Presence or absence of cognitive dysfunction and gender were the qualitative variables and were analysed as frequencies and percentages. Mean ± SD was calculated for all quantitative variables i.e. age, disease duration and years of education.

Data was stratified according to disease duration, into patients with disease duration less than two years (24 months) and disease duration more than two years. Similarly, two more groups were made, one with patients having total duration of formal education of more than 12 years and the other with less than 12 years. For both stratified groups frequency of cognitive dysfunction was calculated and significance was checked by using Chi-square (*x*^2^) test.

## RESULTS

Out of the enrolled 43 patients, 95.3% (n= 41) were females and 4.7% (n= 2) were males. Mean age of patients, disease duration (in months) and years of formal education are shown in Table-I. Anti-nuclear antibodies (ANA) were present in all patients and anti-ds DNA in 93% (n=40) patients.

**Table-I:** Demographic details and disease characteristics (Quantitative variables).

Parameter	Mean ± SD
Age (years)	28.72 ± 9.246
Disease duration (months)	24.21 ± 30.463
Formal Education (years)	10.98 ± 3.291

SD = standard deviation

Cognitive dysfunction according to MoCA score (adjusted for years of formal education) was found in 65.1% (n=28) patients. On stratification of patients according to disease duration, in patients with disease duration more than two years (24 months), cognitive dysfunction was found in 60% patients (9 out of 15) and for those with duration of disease less than two years it was found in 67.8% (19 out of 28). This was not found to be statistically significant according to Chi-square (*x*^2^) test [p>0.05], Table-II. For stratification according to duration of formal education, in patients with less than 12 years of formal education, cognitive dysfunction was found in 74.1% patients (20 out of 27) and for education duration more than 12 years this was 50.0% (8 out of 16). This was also not found to be statistically significant according to Chi-square (*x*^2^) test [p>0.05], Table-III.

**Table-II:** Stratification according to Disease duration with Statistical analysis.

Disease Duration (years)	Frequency (% out of n=43)	Frequency of Cognitive Dysfunction (%)	Frequency of normal Cognition (%)	Chi-square (x^2^) test	p-value
More than 2 years (24 months)	15 (34.9)	9 (60.0)	6 (40)	0.265	0.61 (>0.05)
Less than 2 years (24 months)	28 (65.1)	19 (67.9)	9 (32.1)		

**Table-III:** Stratification according to Years of formal education with Statistical analysis.

Years of formal education	Frequency (% out of n=43)	Frequency of Cognitive Dysfunction (%)	Frequency of normal Cognition (%)	Chi-square (x^2^) test	p-value
More than 12 years	16 (37.2)	8 (50.0)	8 (50.0)	2.563	0.11 (>0.05)
Less than 12 years	27 (62.8)	20 (74.1)	7 (25.9)		

## DISCUSSION

Systemic lupus erythematosus (SLE) is one of the many autoimmune diseases that primarily affect females in their child-bearing age.^15^ SLE causes chronic tissue and organ inflammation through complement activation which is mediated by autoantibodies and immune complexes, this ultimately results in damage to multiple organs.^15^ Cognitive dysfunction is not so uncommon in patients with SLE, and several studies have emphasized on its effect in patients’ health-related quality of life^16^,^17^ and employment.^18^ Neuropsychiatric SLE (NPSLE) has been found to increase morbidity and mortality of SLE patients.^4^

In our study, 43 patients having SLE were evaluated for the presence or absence of cognitive dysfunction using MoCA scale (Urdu version). Our analysis showed that 65.1% (n=28) patients had cognitive dysfunction. There has not been any local or regional study on this topic till date. Cognitive dysfunction has been found to be prevalent in SLE, ranging from 12% to as high as 87% and 95%.^3^,^13^ A study from Egypt used MoCA scale and found that 96.6% of patients showed impairment using MoCA scores, but this study was done in patients having lupus nephritis.^7^ Petri et al. reported the frequency of cognitive impairment as 75%, using the Automated Neurophysiological Assessment Metrics (ANAM).^19^ An Italian study, found prevalence of 31%.^6^ Sanna et al. and Hanly et al. had the lowest reported percentages (11% and 6% respectively);^20^,^21^ but they did retrospective studies.

Our study showed that disease duration has no significant effect on development of cognitive dysfunction; similar results were seen in almost all previous studies even by using different methods of assessment.^7^ Bhasin et al. concluded that cognitive dysfunction in SLE patients has no correlation with duration of illness.^22^ Maneeton et al. also found loss of cognition to be unrelated to disease duration.^23^

When we stratified patients according to education level, we again found no significant cognitive dysfunction in patients with less than 12 years of formal education as compared to those with more than 12 years of formal education. One study concluded that cognitive deficiency is associated with lower education level.^24^ May be this observed difference is due to the difference in the disease dynamics of our population, further studies are recommended to analyse this better.

This study has many advantages. Firstly, MoCA questionnaire is easy to use and interpret and it does not require any special apparatus or expertise. Secondly, it is available is many languages and all have been validated. Lastly, many previous studies have used it, so data can be compared internationally.

### Limitations of the study

Firstly, it was conducted in one centre only and represents a subgroup of patients. To address this limitation a multicentre study can be done in future. Secondly, our study was conducted in a tertiary care teaching hospital where we get referrals from all over Punjab, however, patients at such centres have advanced and complicated disease, so results of this study are better applied for such advanced stage patients and cannot be generalised. Another limitation is that this study was only done for three months and had a size of 43 patients; a bigger and longer study may show different results.

## CONCLUSION

This study concludes that 65.1% (n=28) of SLE patients had cognitive dysfunction and there was no significant difference in cognitive dysfunction in patients according to years of formal education or disease duration. So it is imperative for all clinicians treating patients with SLE to screen for cognitive dysfunction and start treatment early to achieve better outcomes and improve quality of life of SLE patients.
